# Spinal degeneration and lumbar multifidus muscle quality may independently affect clinical outcomes in patients conservatively managed for low back or leg pain

**DOI:** 10.1038/s41598-024-60570-0

**Published:** 2024-04-29

**Authors:** Jeffrey R. Cooley, Tue S. Jensen, Per Kjaer, Angela Jacques, Jean Theroux, Jeffrey J. Hebert

**Affiliations:** 1 College of Science, Health, Engineering and Education, Murdoch University, 90 South Street, Murdoch, WA 6150 Australia; 2 Department of Diagnostic Imaging, Regional Hospital Silkeborg, Silkeborg, Denmark; 3 Spine Centre of Southern Denmark, Middelfart, Denmark; 4 Chiropractic Knowledge Hub, Odense M, Denmark; 5 Department of Sports Science and Clinical Biomechanics, University of Southern Denmark, Odense M, Denmark; 6 Health Sciences Research Centre, UCL University College, Odense M, Denmark; 7 Institute for Health Research, University of Notre Dame Australia, Fremantle, WA Australia; 8 Faculty of Kinesiology, University of New Brunswick, Fredericton, NB Canada

**Keywords:** Musculoskeletal system, Magnetic resonance imaging, Osteoarthritis, Skeletal muscle

## Abstract

Few non-surgical, longitudinal studies have evaluated the relations between spinal degeneration, lumbar multifidus muscle (LMM) quality, and clinical outcomes. None have assessed the potential mediating role of the LMM between degenerative pathology and 12-month clinical outcomes. This prospective cohort study used baseline and 12-month follow-up data from 569 patients conservatively managed for low back or back-related leg pain to estimate the effects of aggregate degenerative lumbar MRI findings and LMM quality on 12-month low back and leg pain intensity (0–10) and disability (0–23) outcomes, and explored the mediating role of LMM quality between degenerative findings and 12-month clinical outcomes. Adjusted mixed effects generalized linear models separately estimated the effect of aggregate spinal pathology and LMM quality. Mediation models estimated the direct and indirect effects of pathology on leg pain, and pathology and LMM quality on leg pain, respectively. Multivariable analysis identified a leg pain rating change of 0.99 [0.14; 1.84] (unstandardized beta coefficients [95% CI]) in the presence of ≥ 4 pathologies, and a disability rating change of − 0.65 [− 0.14; − 1.16] for each 10% increase in muscle quality, but no effect on back pain intensity. Muscle quality had a non-significant mediating role (13.4%) between pathology and leg pain intensity. The number of different pathologies present demonstrated a small effect on 12-month leg pain intensity outcomes, while higher LMM quality had a direct effect on 12-month disability ratings but no mediating effect between pathology and leg pain. The relations between degenerative pathology, LMM quality, and pain-related outcomes appear complex and may include independent pathways.

## Introduction

Because of the lumbar multifidus muscle’s (LMM) contributory role to lumbar spine stability^1–3^, it is generally believed that changes in this muscle groups may have a role to play in the etiology, progression, and/or management of low back pain (LBP) disorders, including LBP-related leg pain and disability. As an association between altered LMM quality and radiculopathy has been noted previously^4,5^, it is possible that nerve root compromise may concurrently result in leg pain and isolated LMM quality reduction. Also, LMM atrophy or reduced functionality have been reported to be associated with limited physical function or disability^6–8^.

Most imaging-based investigations into the LMM’s relationship to low back-related conditions have focused on baseline cross-sectional, cohort, and predictive outcomes. Few non-surgical, longitudinal/prospective studies assessing these relationships have been undertaken. Attempting to synthesize this growing body of evidence, numerous systematic or narrative reviews have looked into the cross-sectional, predictive, or longitudinal relations between the LMM, low-back related clinical outcomes, and/or specific spinal pathologies^9–17^. These reviews have provided further insights into our understanding of the LMM’s potential contribution to LBP. However, clarity of understanding or identification of clear trends that might support or rule out the supposition that the LMM plays an important role in the cause or management of LBP have not been forthcoming.

Longitudinal studies investigating the relationship between LMM morphology and low back-related clinical presentations/outcomes^7,18–22^, have not shown clear associative or predictive relationships between LMM morphology and back or leg pain, particularly after accounting for age, sex, and/or BMI. One study did, however, note an association between increased multifidus cross-sectional area (CSA) and improved 12-month disability outcomes ^7^. Conversely, systematic reviews of investigations reporting on the relationships or prognostic value of LMM morphology with low back pain-related outcomes have identified that relationships may exist^9,10,12,15–17^. However, the results are mixed, and the studies reviewed were primarily non-longitudinal in design.

Additional longitudinal studies have been undertaken to investigate whether long-term relationships exist between lumbar region pathologies and low back-related clinical findings or the ability of pathology to predict clinical outcomes. Specifically, recent studies have reported mixed results, with some identifying degenerative findings as having long-term positive associations with the presence or recurrence of LBP^23–25^. Others report no clinically important long-term associations or predictive capacity between MRI findings and LBP severity, disability, or radicular pain^26–29^, or even inverse associations with LBP activity and disability ratings^30^. One review investigating the ability of MRI findings to predict future LBP identified no consistent associations between MRI findings and future LBP or disability outcomes^13^. However, a follow-on review did identify a predictive capacity between specific combinations of MRI-identified pathologies and responses to different treatments^14^.

No longitudinal studies were identified that assessed for concurrent interactions between the LMM, clinical outcomes, and spinal pathology. Cross-sectional studies looking into these interactions (focused on endplate and/or intervertebral disc changes as the primary pathology variables) reported mixed results^8,31–35^, although most identified significant associative or predictive relationships between these three categories^8,31,34,35^. Bailey et al. further suggested that muscle quality may not be an independent source of a back pain but rather a factor modulating the effect and amount of pain from specific spinal pathologies (e.g., endplate defects)^31^. This led us to postulate a progressive link between lumbar degeneration, LMM quality and future clinical outcomes, wherein degenerative spinal changes may contribute to altered spinal function, resulting in a reduction in muscle quality, which may add to an increased risk of poor recovery^36,37^. Although one narrative review explored the associations between lumbar paraspinal muscles, spinal pathology, and LBP-related outcomes, it did not include studies assessing concurrent interactions between all three categories^11^.

As many of the aforementioned publications indicated a need for additional, well-designed longitudinal studies, our first objective was to estimate the effects of degenerative lumbar spinal pathology and LMM morphology on 12-month pain and disability outcomes in patients conservatively managed for low back pain or back-related leg pain. We hypothesized that patients with lower quality LMM tissue or a greater number of pathologies would demonstrate worse pain and disability outcomes. To test the theory that LMM quality may indirectly mediate pain outcomes, our second objective was to explore the mediating role of LMM morphology in the relationship between lumbar-related degenerative pathology and 12-month pain and disability outcomes. We hypothesized the LMM would partially mediate the effect between the number of lumbar degenerative pathologies and patients’ pain and disability outcomes.

## Material and methods

We conducted a one-year prospective cohort study. The study sample for this project was drawn from patients who presented to the Spine Centre of Southern Denmark between September 2013 to October 2014 with a primary complaint of LBP and/or lower extremity radicular symptoms referred for conservative, non-surgical assessment. Additional eligibility included a completed, prescribed electronic or paper-based clinical history questionnaire (at initial presentation and at 12-month’s follow-up) and available baseline lumbar MRIs from the local hospital radiology department. Patients with pre-coded MRI pathology data were also included, although the absence of coding was not exclusionary. Patients with missing baseline demographic information, presenting with a significant cause of low back pain (e.g., malignancy, infection, recent fracture), missing or undiagnostic images at the L4/5 or L5/S1 spinal levels, imaging with evidence of surgery in the lower lumbar region, or referral for surgical management within the 12-month follow-up period, were excluded.

The baseline and follow-up clinical data and imaging accessed for our study were obtained from the SpineData registry. A full description of the development and scope of the SpineData registry has been previously published^38^. Briefly summarized, this registry contains a collection of patient data that began in 2011 as part of a continuous cohort study approved by the Danish Data Protection Agency for the Region of Southern Denmark (Journal number: 2008–58-0035–15/22,513), performed following the Declaration of Helsinki principles and with signed informed consent from all patients. Danish law does not require further ethical approval from the Regional Committees on Health Research Ethics for Southern Denmark to access this data (a letter of exemption is available in Danish from the authors on request). Approval for the inclusion of this data for analysis within a larger project was provided by Murdoch University’s Human Research Ethics Committee (approval: 2017/110).

### MRI acquisition protocols

The MRIs used in this study were obtained using a body/spine coil on either a 1.0 T Philips Panorama (Best, The Netherlands) or 1.5 T Philips Achieva (Best, The Netherlands) MRI system. Sagittal T1- and T2-weighted turbo spin echo (TSE) and axial T1- and T2-weighted TSE sequences (angled along the L3/4 – L5/S1 disc planes) were acquired. All sequences were used for the pathology coding process. The axial T2-weighted TSE MRI sequences were used for LMM analysis, with T1-weighted sagittal imaging used to assist with spinal level localization.

### Pathology selection and assessment criteria

Included cases had been sequentially selected to code MRIs for the presence and characteristics of any spinal pathology, from which we included degenerative pathologies identified between L2/3 and L5/S1. These pathologies included disc degeneration (Pfirrmann grading), bulges, high intensity zones, herniations (including any associated nerve root compromise), Modic marrow changes, endplate defects, vertebral osteophytes, and facet arthrosis. If stenosis was present, it was assessed within its degenerative cause (i.e., herniation, bulge, arthrosis) rather than as a standalone finding. Pathology coding was performed blinded to the clinical data. To investigate the effect of multiple, concurrent degenerative findings, we categorized the above listed pathologies into 3 groups, based on the number of pathologies present rather than specific pathology combinations [see Table 1]. Disc bulges, high intensity zones, endplate defects, and osteophytes were considered present if they were found at at least one spinal level. The remaining pathologies were considered present if they were coded as moderate to severe in nature. A full description of the pathology selection, coding, and aggregate analysis protocols, has been reported^39^.

**Table 1:** Descriptive statistics: demographic, muscle, pathology, and clinical variables.

Variables		Total N	
Age (years, at 1st visit)		569	45.1 (9.7)
BMI (kg/m^2^)		569	26.8 (4.9)
Female sex		569	334 (58.7%)
Current symptom duration		564	
	< 3 months		121 (21.5%)
	3–12 months		229 (40.6%)
	> 12 months		214 (37.9%)
Average % MCSA		569	33.9 (11.7)
Number of pathologies present		358	
	< 2		74 (20.7%)
	2–3		129 (36.0%)
	≥ 4		155 (43.3%)
Low back pain intensity (0–10)			
Baseline		568	5.8 (2.2)
12-month		568	4.4 (2.7)^1^
Leg pain intensity (0–10)			
Baseline		568	4.5 (2.9)
12-month		568	3.0 (2.8)^1^
Low back pain-related disability (0–23)			
Baseline		558	13.5 (5.3)
12-month		558	9.1 (6.7)^1^

Values in right column represent mean (SD), or counts (%); % MCSA = proportion of peak muscle cross-sectional area.

^1^*P* < 0.001 from baseline.

### Muscle measurement parameters

All LMM measures were acquired bilaterally at the L4/5 and L5/S1 disc levels, using the image slice providing the clearest posterior arch anatomy and LMM outlines at each level. Measures were performed by the lead author, who was blinded to the clinical and coded pathology data, using sliceOmatic v5.0.8b [TomoVision, Magog, Canada]. Histogram analysis of the whole image was then used to determine the cut-off signal value between tissue types, with darker tissues (e.g., muscle) predominating at the lower end of the histogram scale. Muscle assessment for this study focused on quality (i.e., the “pure” muscle component) rather than quantity (i.e., the total muscle area)^40^. To provide a reproducible estimate of muscle tissue quality, the maximum muscle signal intensity peak (“peak muscle signal”) within an image histogram was identified and set as the muscle cut-off value.

**Figure 1:**
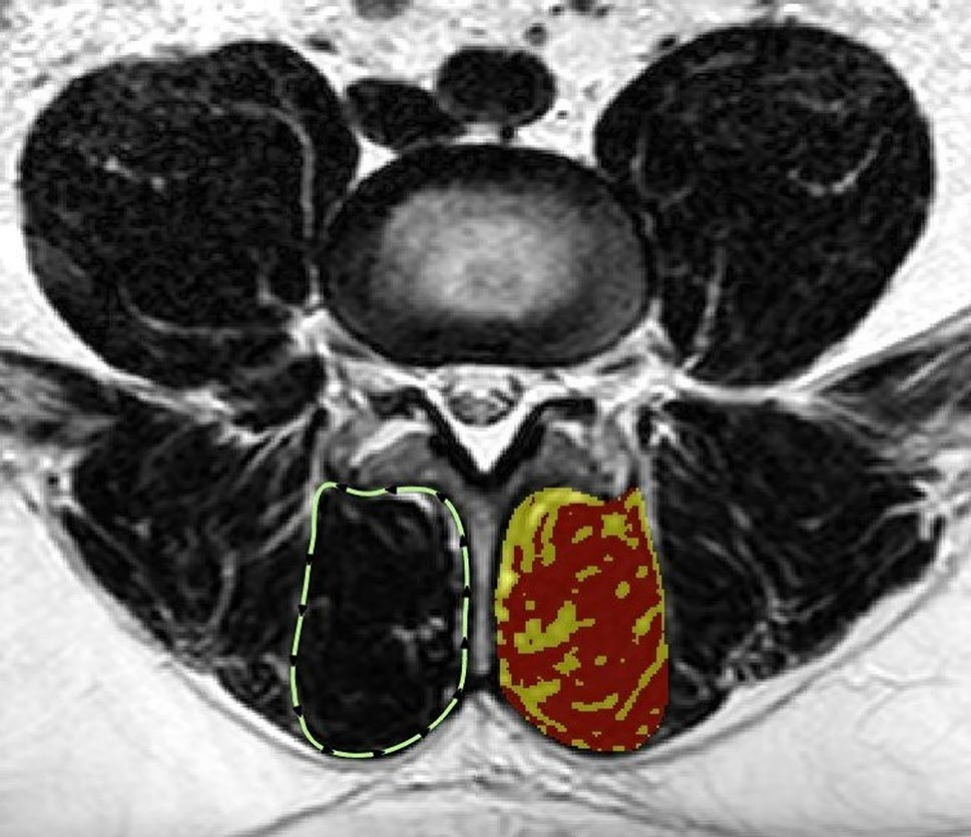
Muscle measurement method. Total CSA outlining of multifidus muscle (dotted line on the left); example of pure muscle component highlighting (red regions on the right).

The LMMs were outlined using protocols applied previously to determine the total CSA (Fig. 1)^41^ . The “peak muscle signal” was applied to the total CSA to separate the higher quality muscle from the remaining tissues. The proportion (from 0.0 – 1.0) of peak muscle CSA present was then calculated [peak muscle CSA (cm) / total CSA (cm) = proportion muscle CSA] and reported as the total peak muscle CSA percentage (% MCSA) (Fig. 1). Next, the average % MCSA was calculated from the four multifidus muscle measures acquired at L4/5 and L5/S1 bilaterally. The complete details of the image selection process and LMM measurement protocols have been previously reported^39^, but it is important to note that the % MSCA value is based on a reproducible estimate of muscle quality and is not meant to reflect a precise measure of LMM health.

### Demographic and clinical details

#### Baseline data

Details regarding the duration of symptoms were collected. The duration of symptoms was based on patients’ current presentation only, not any prior pain history. Additionally, the following pain characteristics and LBP-related disability measures were collected: 11-point (0–10) numeric pain scales (NPS) to separately quantify LBP intensity, including the buttocks, and leg pain intensity (calculated from the average of the current pain rating and the typical and worst pain ratings over the preceding 14 days)^42^; and, patient-rated LBP-related disability, using the 23-item version (0–23 scale) Roland Morris Disability Questionnaire (RMDQ)^43^.

#### Treatment and follow-up data

Following completion of an initial clinical work-up, patients were referred for conservative, non-invasive case management by a Spine Centre clinician. This could be within the Spine Centre, or externally for community-based care. Conservative management could include, either separately or in combination, manual therapy (e.g., chiropractic, osteopathy), rehabilitation or prescribed therapy (e.g., physiotherapy, exercise therapy), acupuncture, oral pain medications (medically prescribed or OTC), or patient selected options (e.g., Pilates, swimming, psychology). Patients were able to continue, discontinue, and/or re-establish their care at their own discretion; however, irrespective of type or length of conservative treatment, at 12 months following their initial presentation all patients were invited to provide follow-up pain intensity and disability ratings information (as described under baseline data). It should be noted that the specific types and lengths of treatment received during the intervening 12 months were not assigned by the researchers, but formed part of the usual care provided within this healthcare system as determined by the clinical staff and each patient. As such specific treatment information was not collected or analyzed within this study.

**Figure 2:**
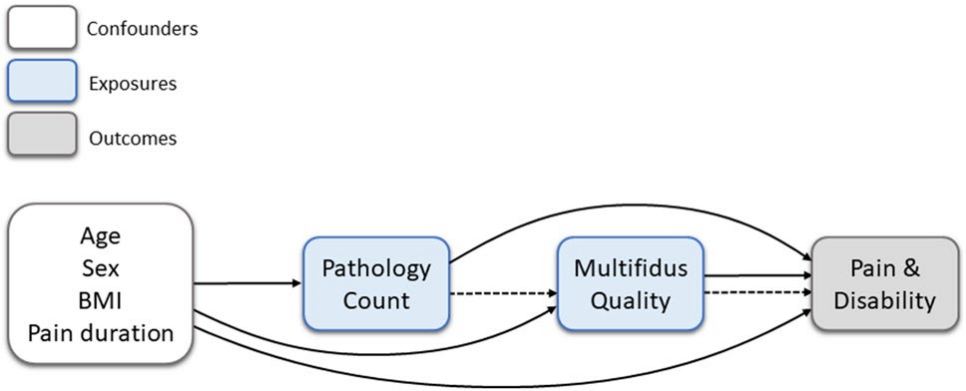
Overview of relationships between confounder, exposure, and outcome variables. Solid arrows = direct analysis pathways; dashed arrows = mediation analysis pathway.

Self-reported baseline age, sex, height (cm) / weight (kg) (for Body Mass Index (BMI) calculation), and the length of time since current low back or leg pain onset (recorded to the nearest month, with “0” being used for pain of less than one month’s duration), were included as confounders. The relationships between the confounders and other variables are overviewed in Fig. 2.

### Statistical methods

To estimate the effect of degenerative pathology and LMM quality on 12-month clinical outcomes, we constructed generalized linear mixed effects models with random-intercepts to account for patient heterogeneity, and with all other variables as fixed effects. Mixed effects tobit models were used for pain outcomes with ceiling or floor effects, and linear mixed models were used for RMDQ outcomes. We modelled each exposure (muscle quality, spinal pathology) and outcome (low back pain intensity, leg pain intensity, and low back pain-related disability), in separate models and adjusted for the confounders age, sex, BMI, and pain duration at baseline. We then explored the mediating role of LMM quality for the one significant model for degenerative spinal pathology (i.e., leg pain) as follows: (i) regressing leg pain on pathology count (total effect); (ii) regressing LMM quality on pathology count (partial effect); and (iii) regressing pathology count on leg pain (direct effect) and LMM quality and pathology count on leg pain (indirect effect). Results were summarised using beta coefficients and 95% confidence intervals. During mediation analysis, we accounted for age, sex, BMI, and pain duration between exposure and mediator as well as between mediator and outcome. The significance of the indirect effect coefficient was tested by generating a 95% confidence interval using bootstrapping. Hypotheses were two-sided, and significance levels for all analyses were set at α = 0.05. As this was an exploratory study and we wanted to avoid missing potentially real differences, we elected not to modify alpha to account for the multiple tests. All data were analyzed using Stata I/C version 17.0 (StataCorp, College Station, TX).

We conducted sensitivity analyses for each significant model to quantify the potential impact of unmeasured confounding. We calculated E-values^44,45^ for the parameter estimate and the lower bound of its confidence interval, using one standard deviation of the average % MCSA for the value for muscle quality analysis. The default contrast of interest in exposure value of 1 was applied for pathology analysis. The E-value estimates the magnitude of an unmeasured confounder’s association (on the risk ratio scale) with both the exposure and the outcome needed to explain away the results (E-value for the parameter estimate) or to include the null value (E-value for the confidence interval).

## Results

**Figure 3:**
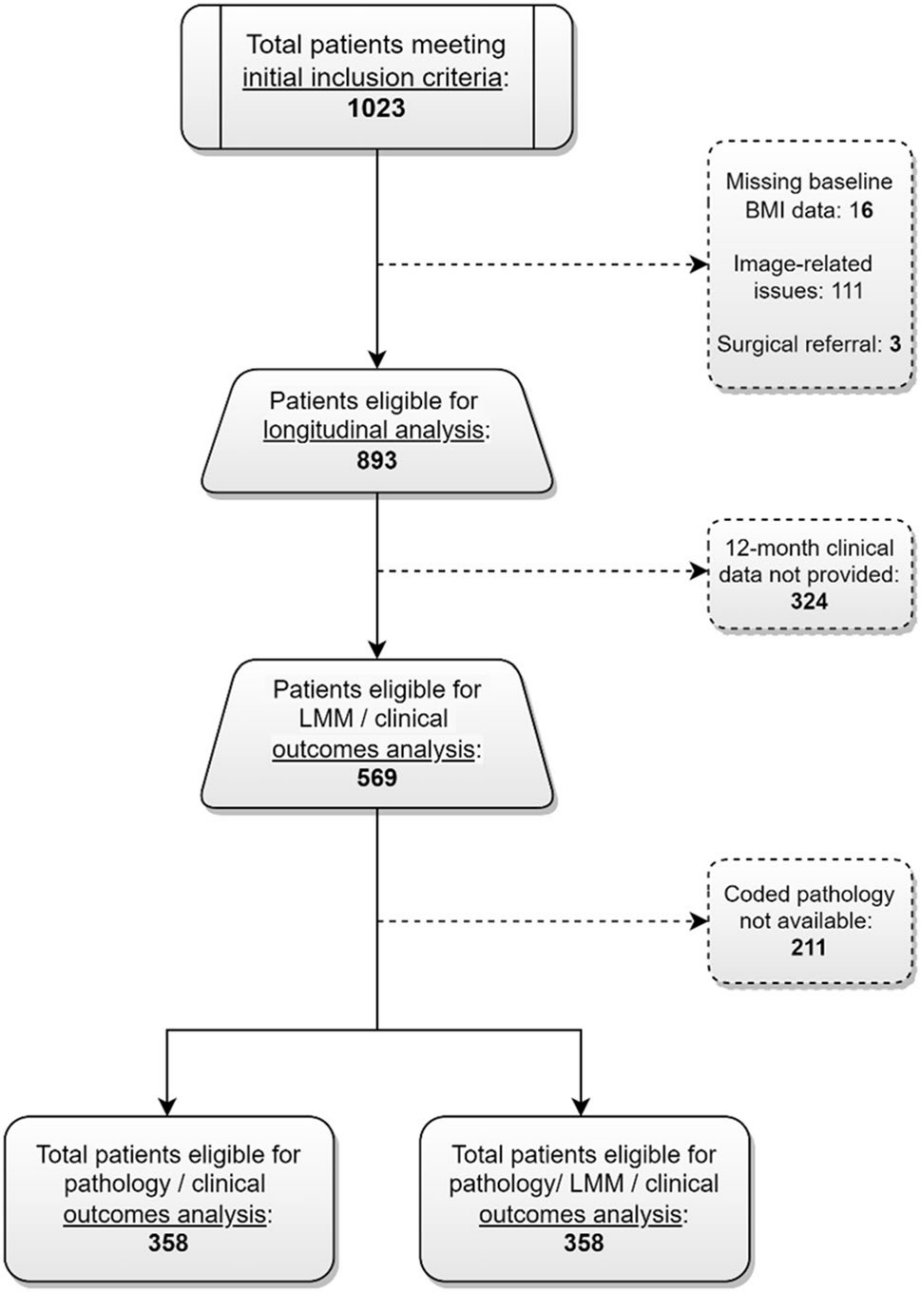
Case selection flowchart.

The patient selection process is presented in Fig. 3. From the pool of patients eligible for longitudinal analysis, 569 (64%) provided 12-month follow-up data for inclusion in the LMM / clinical outcomes analysis. Of those patients with follow-up clinical data, 358 (63%) also had coded data available to perform the pathology-related analyses. If patients did not provide input for a specific clinical variable (e.g., no baseline or 12-month rating), this resulted in a further reduction in the number of evaluated cases for that variable.

The mean (SD) age was 45.1 (9.7) years, with 59% of patients being female. Fourteen participants (2.4%) had leg pain without current LBP; 190 (34%) demonstrated ≥ 30% improvement in their RMDQ score at 12-month follow-up (< 3 months: 56 (46%); 3–12 months: 94 (41%); > 12 months: 40 (19%)). Additional descriptive data is provided in Table 1.

### Effects of baseline muscle quality on 12-month clinical outcomes

We identified a significant effect of muscle quality on pain-related disability, with a 10% higher average % MCSA associated with 0.65 fewer points on the Roland-Morris score (95% CI = -0.14 to -1.16; *p* = 0.010). That is to say, patients with a higher proportion of healthy muscle at baseline showed greater improvement in their disability ratings at 12 months. There was no apparent effect of a 10% higher average % MCSA on 12-month leg pain intensity or low back pain intensity, although the former was close to our threshold of statistical significance (β [95% CI] = -0.23 [-0.48 to 0.01] (*p* = 0.058)) (Table 2).

**Table 2:** Estimated effects of muscle morphology and degenerative spinal pathology of low back and leg pain intensity and low back pain-related disability.

Predictor	Outcome		Multivariable
		N	β (95% CI) [*p-value*]
Average % MCSA	LBP intensity	568	− 0.11 (− 0.32 to 0.09) [*0.309*]
	LP intensity	568	− 0.23 (− 0.48 to 0.01) [*0.058*]
	Disability	558	− **0.65 (**− **1.16 to **− **0.14) [*0.010*]**^**1**^
No. of pathologies	LBP intensity	357	
< 2			Ref
2–3			0.30 (− 0.41 to, 1.01) [*0.439*]
≥ 4			− 0.02 (− 0.74 to 0.70) [*0.898*]
	LP intensity	357	
< 2			Ref
2–3			0.67 (− 0.17 to 1.50) [*0.126*]
≥ 4			**0.99 (0.14 to 1.84) [*0.025*]**^**1**^
	Disability	351	
< 2			Ref
2–3			0.94 (− 0.82 to 2.70) [*0.338*]
≥ 4			0.78 (− 1.00 to 2.56) [*0.459*]

Outcomes based on baseline to 12-month change; all models adjusted for patient age, sex, body mass index, and pain duration at baseline.

*β* beta coefficient, *CI* confidence interval, *% MCSA* proportion of peak muscle cross-sectional area, *LBP* low back pain, *LP* leg pain. ^1^Statistically significant result.

### Effects of baseline degenerative spinal pathology on 12-month clinical outcomes

A significant effect of spinal pathology on leg pain intensity was noted, with the presence of 4 or more aggregate pathologies being associated with a 0.99-point increase in leg pain ratings (95% CI = 0.14 to 1.84; *p* = 0.025) when compared to having one or no degenerative lumbar pathologies. No other significant findings were identified (Table 2).

### Mediation of degenerative spinal pathology by muscle quality

**Figure 4:**
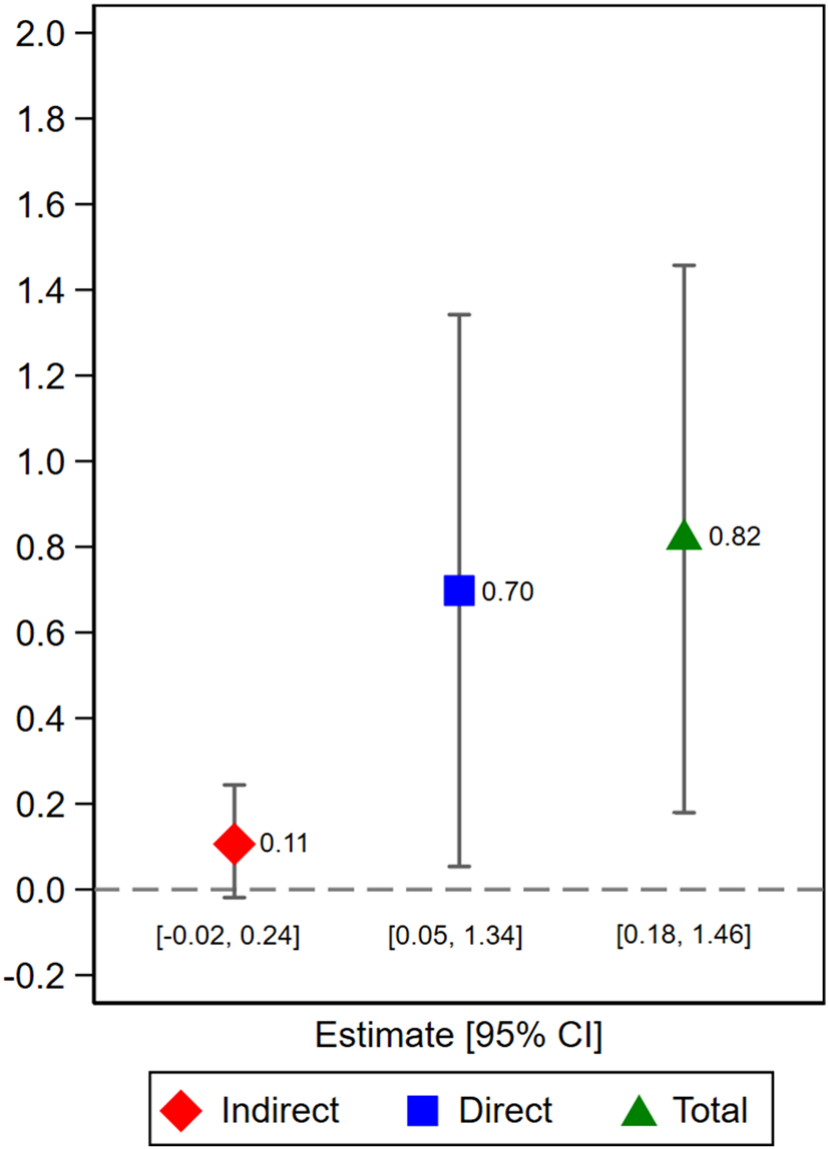
LMM mediation. Indirect effect represents the contribution of change in leg pain intensity attributable to the LMM; direct effect represents the contribution attributable to pathology.

Effect decomposition identified a non-significant (13.4%) mediating role of LMM quality between degenerative spinal pathology and 12-month leg pain intensity, with the direct effect accounting for 85.4% of any change (Fig. 4).

### Sensitivity analyses

Given the effect of muscle quality on low back-related disability, unmeasured confounding of 1.46 or above on the risk ratio scale, beyond the measured confounders, could explain away the estimate, but a weaker level of confounding could not. Similarly, unmeasured confounding at or above 1.18 on the risk ratio scale could shift the confidence interval to include the null, but weaker confounding could not. For pathology, given the effect of the number of degenerative pathologies on leg pain intensity, unmeasured confounding of 2.09 or above, beyond the measured confounders, could explain away the estimate, but a weaker level of confounding could not. Likewise, unmeasured confounding at or above 1.27 on the risk ratio scale could shift the confidence interval to include the null, but weaker confounding could not.

## Discussion

This study aimed to investigate the longitudinal relationships between degenerative spinal pathology, LMM quality, and clinical outcomes related to LBP, leg pain, and disability, and for any mediating role of the LMM. We partially confirmed our first hypothesis, demonstrating that a greater proportion of higher quality LMM appears to contribute to small reductions in disability ratings at 12 months, while a greater number of different lumbar region degenerative pathologies appears to contribute to small increases in leg pain intensity at 12 months. Sensitivity analysis estimating the effect of any unmeasured confounder(s) between LMM quality and disability ratings, and between pathology and leg pain ratings, indicated that a moderate level of additional confounding might explain away the estimate or shift the confidence interval to include the null for both comparisons.

Regarding LMM quality and disability, we included the most common confounders assessed in the related literature, but due to data limitations, we were unable to measure for potential confounding relating to physical activity. However, the role of physical activity in LMM quality and disability analysis is unclear, with recent studies indicating that physical activity in general was not a confounder for, or associated with, LMM morphology in adults or children with LBP^18,20^. No studies investigating different levels of physical activity as a confounder between LMM morphology and disability were identified. As such, physical activity in general appears unlikely to explain away the effect of LMM quality on disability. In relation to pathology and leg pain, a possible unmeasured confounder would be significant prior trauma. Although we did not have access to this historical information, patients with evidence of spinal fracture were not included, reducing the likelihood of this variable explaining away the effect of pathology on leg pain found in our analysis.

Neither LMM quality nor the cumulative effect of degenerative pathologies affected LBP intensity, and there was no cumulative pathology effect on 12-month disability outcomes. This limited the analysis of our second hypothesis to leg pain outcomes, with the LMM demonstrating a small and non-significant mediating role between degenerative pathologies and changes in leg pain intensity. As such, our second hypothesis was not supported.

The absence of a relationship between LMM quality and changes in LBP intensity found in our study agrees with findings from other longitudinal studies looking at a diverse collection of muscle and LBP outcome parameters. These have included comparisons of: intramuscular fat in childhood at L4 and L5 (combined) with the likelihood of developing LBP in early adulthood^18^; the CSA, functional CSA, intramuscular fat, and muscle asymmetry at L3/4 and L5/S1 with changes in frequency and intensity of LBP^19,20^; the highest percentage of intramuscular fat at L4 or L5 as a predictor of future LBP^21^; the CSA at L3 and L5 as a prognostic indicator of LBP chronicity^22^; and, the CSA at L3, L4, and L5 as predictors of LBP intensity^7^. These longitudinal studies have evaluated over 1,400 participants from Finnish, Danish, and Korean populations, comparing the standard static measures of L3/4 through L5/S1 LMM morphology to a wide variety of LBP outcome measures, with none demonstrating predictive, prognostic, or causative relationships between LMM morphology and LBP outcomes. This suggests that future research may be more effectively directed towards other outcome measures (e.g., leg pain, low back-related disability), muscle measures (e.g., dynamic/functional), or treatment outcomes. Assessing other populations would help determine if this lack of relationship with LBP is regional versus global in nature.

Our finding of higher LMM quality potentially contributing to reduced leg pain intensity is unique to other longitudinal studies. Two related studies published by Fortin et al. ^19,20^, noted adjusted associations between the presence of sciatica and LMM asymmetry (but not for LMM quantity or quality) at 15-year follow-up, while Hebert et al. ^21^, found an adjusted cross-sectional association between the severity of LMM fat infiltration and ever having had leg pain at baseline measure, which did not persist at 5- or 9-year follow-up. While the timelines assessed varied substantially between studies (1 year for our study versus 5–15 years for the others), it is more probable that the difference in outcomes is related to the leg pain assessment format applied. Our study assessed leg pain intensity ratings, whereas Fortin and Hebert assessed dichotomous “presence/absence of leg pain” outcomes.

An inverse relation between LMM quality and changes in low back-related disability was consistent with findings from Ranger et al.^7^. Although their study evaluated a similar patient population, the spinal levels evaluated and muscle assessment methods applied between studies varied. Regardless, both studies found that LMM quality and quantity may contribute to changes in low back disability (i.e., the presence of either greater muscle size or a greater proportion of higher quality muscle may contribute to lower disability ratings at 12 months).

In testing our theory of an indirect LMM pathway between pathology and clinical outcomes, we found that the LMM does not appear to contribute a mediating role between pathology and pain or disability outcomes, even though LMM quality and aggregate degenerative pathologies separately demonstrated an effect on leg pain outcomes at one-year follow-up. We had speculated that spinal pathologies in general may affect spinal function, thus altering LMM quality, with both changes contributing to leg pain. Instead, there may be a more localized connection between compromise of the nerve root supplying the LMM, LMM quality, and leg pain intensity. Regardless, the noted results suggest that whatever effect muscle quality and degenerative changes have on leg pain outcomes, those effects appear to occur independently.

The findings from this and other longitudinal studies indicate that altered LMM morphology may play a role in the development of low back-related leg pain and disability. However, the relevance of these findings would be moot if improvements in multifidus muscle quality and clinical outcomes were not also achievable. Kalichman et al.’s 2017 narrative review provides some insight into this question, identifying several studies which demonstrated the potential for intensive spinal exercise programs to contribute to short-term stabilization or even reversal of paraspinal muscle degenerative changes (i.e., improved muscle strength, density, or thickness/CSA) and outcomes in different LBP subgroups^11^. More recent randomized clinical trials in patients with LBP have confirmed that focused exercise programs not only improved LMM morphology in the short term but were associated with better pain or disability outcomes versus patients without muscle improvement^46,47^. Assessing whether these changes may be further impacted by specific subgroups (e.g., the presence of minor versus severe spinal degeneration, or few versus several degenerative pathologies), as well as whether there is long-term persistence of improved LMM and clinical outcomes, would be important areas of focus for ongoing LMM research. And while the confounding effect of physical activity in general is questionable, the potential for specific exercise regimes to have a confounding effect could be a further research consideration.

Incidentally, we noted significant improvement in clinical outcomes between baseline and 12-month measures (Table 1). Given the tendency for improvement of pain and disability ratings in a population of people, from natural history and/or conservative interventions, this was not an unexpected outcome. Nevertheless, neither LMM quality nor the amount of pathologies present appeared to play a role in improving LBP intensity ratings.

Our study had several strengths*,* including a larger sample size and clinical, muscle, and pathology data comparisons utilizing 12-month clinical outcome data and a standardized assessment process for degenerative spinal pathology. There were also some limitations. We experienced a loss of 12-month follow-up responses for 36% of patients. As there were only two time points for outcomes collection, we chose to apply a complete case analysis for this study. This increased the possibility of selection bias. The pathology coding process included a sequential selection of the first half of presenting cases only, which may also have contributed to selection bias. However, as the patients included presented over a 6-month period, it is unlikely any particular degenerative pathology categories would have been emphasized or excluded. This article represents the final component of a multi-phase project, initiated in 2013, with clinical data being collected over a two-year period as part of the original study design. As the SpineData database has been discontinued and data has been anonymized, it was no longer possible to merge more recent patient data with MRI scans. Nevertheless, we believe the relevance of the initial data has not dissipated, but represents a snapshot in time of a multi-year cohort study within the same general population. Further, although we did have data relating to patients’ current pain duration, we did not have information pertaining to any previous history of low back or leg pain. As such, we could not consider the impact of any previous pain episodes when analyzing outcomes against pain duration. While we did have information regarding prior surgical treatment, no information was available regarding the presence, type, and/or frequency of conservative therapies prior to referral to the Spine Centre. However, as this project was not focused on outcomes related to specific therapies, and there were 12 intervening months between our baseline and follow-up measures, any meaningful impact from prior conservative management on the results of this study is expected to be small. Concurrently, as this study aimed to investigate the effects of pathology and muscle quality on clinical outcomes in conservatively managed patients, rather than to assess the effect of any specific conservative treatment, any confounding effect specific treatment protocols may have had on our outcome measures is unknown. This may serve as a focus for future studies in this area of research. Finally, although LMM quality was associated with a statistically significant change in disability ratings, the level noted was below the reported minimal clinically important difference (MCID) for our instrument of 2.0, which may limit the clinical importance of this change^43^.

Although not specific to our study, a common limitation with imaging studies of LMM morphology is a lack of standardization in the depiction and description of LMM morphology^48^. Varying sample sizes, age and BMI of participants, quality of studies, and the procedures used to measure fat infiltration have also been suggested as possible reasons for inconsistencies in results of previous studies^49^. However, it is also likely that the complex and potentially multi-directional relationships between pathoanatomical, functional, and clinical outcomes are contributing to the ongoing variances/ inconclusive outcomes being published. While the latter issues can be addressed with ongoing research, to address the issues of LMM measurement inconsistency, we followed recently recommended muscle CSA outlining protocols^50^. A further limitation of all observational studies is the potential for unmeasured confounding. In addition to controlling for all common confounders, we also quantified the potential impact of unmeasured confounding through sensitivity analyses.

## Conclusions

In a secondary spinal care setting, multifidus muscle quality and the number of different degenerative pathologies present both demonstrated small, independent relations with 12-month leg pain ratings outcomes, while higher LMM quality was also related to a small reduction in 12-month disability ratings. Neither LMM quality nor degenerative pathologies demonstrated relations with 12-month LBP rating changes, and LMM quality did not play a significant mediating role between pathology and leg pain outcomes. The relations between spinal degeneration, multifidus muscle degeneration, and LBP-related outcomes appear complex and may include independent pathways. Research identifying patients who would benefit from treatments for LBP-related findings, which account for specific underlying pathoanatomical degenerative processes, is recommended.

## Data Availability

The imaging and clinical datasets analyzed during the current study are not publicly available as they are patient files covered by EU privacy legislation, requiring permission from the database manager to access. Upon reasonable request to the corresponding author, deidentified datasets generated from this study may be made available with database manager approval.

## Abbreviations

% MCSA: Percentage of peak muscle cross-sectional area
BMI: Body mass index
CSA: Cross-sectional area
LBP: Low back pain
LMM: Lumbar multifidus muscle
RMDQ: Roland Morris Disability Questionnaire
